# Non-Thyroidal Illness Syndrome and Thyroid Autoimmunity in Hospitalized COVID-19 Patients: A Retrospective Study

**DOI:** 10.3390/jcm14196784

**Published:** 2025-09-25

**Authors:** Ewa Kozłowska, Milena Małecka-Giełdowska, Olga Ciepiela

**Affiliations:** 1 Central Laboratory, Central Teaching Hospital of University Clinical Center, Medical University of Warsaw, 02-097 Warsaw, Poland; milena.malecka@wum.edu.pl; 2 Department of Laboratory Medicine, Medical University of Warsaw, 02-097 Warsaw, Poland

**Keywords:** COVID-19, non-thyroidal illness syndrome, thyroid function, thyroid autoantibodies, reverse T3, fT3/rT3 ratio

## Abstract

**Background**: Thyroid dysfunction, including non-thyroidal illness syndrome (NTIS), is commonly observed in critically ill patients and has been reported in COVID-19, particularly in those with severe disease. NTIS is defined by low free triiodothyronine (fT3) with normal or low thyroid-stimulating hormone (TSH) and free thyroxine (fT4) levels. Thyroid autoantibodies may also reflect immune system activation. The relationship between thyroid hormone alterations, autoimmunity, and clinical severity in COVID-19 remains incompletely understood. **Methods:** We conducted a retrospective study of 276 patients hospitalized with COVID-19, including 138 in the intensive care unit (ICU) and 138 in general wards. A control group of 110 hospitalized, non-infected patients was also analyzed. Serum concentrations of TSH, fT3, fT4 and reverse T3 (rT3) were measured. The presence of anti-thyroid peroxidase (anti-TPO), anti-thyroglobulin (anti-Tg), and thyrotropin receptor antibodies (TRAb) was assessed. **Results:** NTIS was observed in 44.2% of ICU patients, 18.1% of non-ICU patients, and 1.8% of controls. The fT3/rT3 ratio was lowest in ICU patients (median 0.11 vs. 0.16 in non-ICU and 0.22 in controls). Thyroid autoantibodies were significantly more prevalent in COVID-19 patients than in controls, with anti-TPO antibodies being the most frequently detected. Their presence, even in patients without known thyroid disease, may reflect immune activation associated with SARS-CoV-2 infection. **Conclusions:** NTIS and thyroid autoimmunity are frequent in hospitalized COVID-19 patients and may reflect disease severity and immune activation. Our study highlights the prognostic relevance of routine thyroid testing, including the fT3/rT3 ratio and combined autoantibody positivity (notably the triple-positive pattern), by directly comparing ICU and non-ICU patients with a non-COVID control group.

## 1. Introduction

Since the outbreak of the COVID-19 pandemic caused by the SARS-CoV-2 virus, researchers have actively sought to identify risk factors that influence the severity and prognosis of the disease. While initial investigations primarily focused on comorbid conditions such as cardiovascular diseases, diabetes, and chronic respiratory disorders, accumulating evidence suggests that endocrine dysfunctions—particularly thyroid abnormalities—may also significantly impact the clinical course of COVID-19 [1]. COVID-19 has also been linked to a spectrum of autoimmune complications involving multiple organs [2].

The thyroid gland plays a vital role in regulating systemic metabolism, cardiovascular homeostasis, and immune function through the secretion of triiodothyronine (T3) and thyroxine (T4). Approximately 95% of hypothyroidism and 50% of hyperthyroidism cases are of autoimmune origin, involving diseases such as Hashimoto’s thyroiditis and Graves’ disease [3]. Both hypo- and hyperthyroidism can disrupt systemic inflammatory pathways and modulate the immune response of the host organism. Hypothyroidism has been associated with impaired cellular and humoral immunity, while hyperthyroidism may exacerbate cardiovascular instability in acute illness [4].

Critically ill patients often demonstrate biochemical features of non-thyroidal illness syndrome (NTIS) [5], also known as low T3 syndrome or euthyroid sick syndrome. NTIS is characterized by low serum T3 levels, elevated reverse T3 (rT3), and normal or low T4 concentrations, usually without an increase in TSH [6]. Although traditionally considered an adaptive response to conserve energy during acute illness, persistent NTIS may reflect a maladaptive dysregulation of the hypothalamic–pituitary–thyroid (HPT) axis, associated with poor clinical outcomes [7].

In patients with COVID-19, thyroid dysfunction may arise from multiple factors: direct viral invasion of thyroid tissue, cytokine storm-induced inflammation, effects of therapeutic agents (e.g., glucocorticoids or heparin), and the unmasking or triggering of thyroid autoimmunity [8]. Post-mortem studies and clinical reports have shown evidence of subacute thyroiditis and thyroid hormone disturbances in patients with SARS-CoV-2 infection [6]. Subacute thyroiditis has additionally been reported following SARS-CoV-2 vaccination [9]. In addition to thyroid autoimmunity, COVID-19 has been linked to extra-pulmonary autoimmune manifestations such as concurrent myositis and myocarditis [10], systemic immune-related disorders including vasculitis [11], and latent autoimmunity characterized by an increased frequency of thyroid and other autoantibodies [12].

Importantly, abnormal thyroid function tests (TFTs) have been widely reported in hospitalized COVID-19 patients. The prevalence of NTIS appears to be particularly high among those admitted to intensive care units (ICU) and has been proposed as a predictor of mortality [13]. Several recent multi-centre cohorts confirm that abnormal TFTs correlate with radiological severity and in-hospital death [14]. Furthermore, elevated levels of thyroid autoantibodies, especially anti-thyroid peroxidase (anti-TPO) and anti-thyroglobulin (anti-Tg), have been detected in a substantial proportion of COVID-19 patients, suggesting an underlying autoimmune mechanism or reactivation of latent thyroiditis [15]. Transient low T3 syndrome has been shown to predict progression to critical COVID-19 [16]. Mechanistic insights into thyroid autoimmunity in COVID-19 have been comprehensively reviewed by Fallahi et al. [17].

Despite these observations, the relationship between thyroid hormone disturbances, autoimmune reactivity, and COVID-19 severity remains incompletely understood. Few studies have directly compared ICU and non-ICU patients in terms of thyroid function and antibody profiles.

The primary objective of this study was to evaluate the relationship between thyroid function and the course of COVID-19 in patients treated in intensive and non-intensive care units. In addition, the presence of previously undetected thyroid dysfunction was assessed in the study participants. We analyzed thyroid function and thyroid autoantibody test results to explore the impact of SARS-CoV-2 infection on thyroid status during the acute phase of the disease.

## 2. Materials and Methods

### 2.1. Study Population

We retrospectively extracted data from the electronic medical records for adult patients hospitalized with laboratory-confirmed COVID-19 at the Central Teaching Hospital of the University Clinical Centre of the Medical University of Warsaw between March 2020 and December 2021, covering several pandemic waves. Variant-specific effects could not be assessed, as genomic sequencing was not routinely available at that time. The study included consecutive adult patients with laboratory-confirmed COVID-19 hospitalized either in the intensive care unit (COVID ICU), or in other hospital units (COVID non-ICU), provided that a complete panel of thyroid function tests including autoantibodies was available and no history of thyroid disease was reported. This resulted in 138 patients in each COVID-19 subgroup (82 men and 56 women; mean age 68 ± 15 years and 84 men and 54 women; mean age 69 ± 18 years, respectively). The control group (*n* = 110) consisted of hospitalized patients admitted for non-infections conditions (mainly surgical, cardiological, and neurological defined by the absence of clinical symptoms such as fever, cough, sore throat, or runny nose), with procalcitonin < 0.5 ng/mL and negative SARS-CoV-2 PCR, and no history of thyroid disease, and with complete thyroid testing (80 men and 30 women, mean age 52 ± 18 years).

Thyroid function tests and autoantibodies were obtained at hospital admission or within the first 24 h of hospitalization, and the first available measurement was used for analysis. This retrospective study included analysis of TSH, thyroid hormones (fT3, fT4), reverse T3, and thyroid autoantibodies: anti-thyroid peroxidase (anti-TPO), anti-thyroglobulin (anti-Tg), and thyrotropin receptor antibody (TRAb). Serum TSH, fT3, and fT4 levels were measured using electrochemiluminescence immunoassays (Cobas e801, Roche Diagnostics, Mannheim, Germany). The reference ranges were: TSH 0.27–4.20 μIU/mL, fT3 2.0–4.4 pg/mL, and fT4 0.92–1.68 ng/dL. Thyroid autoantibodies were measured using Roche chemiluminescence immunoassay kits (Hoffmann-La Roche AG, Basel, Switzerland). Positive results were defined as: anti-TPO > 34 IU/mL, anti-Tg > 115 IU/mL, and TRAb > 1.75 IU/L. Reverse T3 was measured using chemiluminescence immunoassay (Maglumi X3, Snibe Diagnostics, Shenzhen, China), with a reference range of 0.31–0.95 ng/mL. Patients with rT3 results were stratified into groups with low and normal fT3 levels.

### 2.2. Statistical Analysis

Statistical analyses were performed using GraphPad Prism 10 software (GraphPad Software, San Diego, CA, USA). The Shapiro–Wilk test was used to assess data distribution. Nonparametric data were analyzed using the Kruskal–Wallis test. The Mann–Whitney U test was used for comparisons between the COVID-ICU and COVID non-ICU groups, as well as between patients with low and normal fT3 levels. Continuous variables were expressed as median values with interquartile ranges (IQR), defined as the first quartile (Q1, 25th percentile) and third quartile (Q3, 75th percentile).

Categorical variables were compared using the chi-square (χ^2^) test. When expected cell counts were below five, Fisher’s exact test was applied instead. These tests were used to evaluate differences in the occurrence of thyroid autoantibody variants among all three study groups (ICU, non-ICU, and control), as well as to assess differences in the frequency of thyroid function disturbances between ICU and non-ICU patients.

Predictor analysis was based on univariate testing of all available baseline demographic, clinical, and thyroid parameters for their association with thyroid autoantibody positivity. Variables with complete data and clinical relevance were retained for further evaluation.

Results were considered statistically significant at *p* < 0.05. No formal correction for multiple comparisons was applied, as the analyses were exploratory in nature, aiming to identify potential associations rather than to test a single predefined hypothesis.

## 3. Results

**Figure 1:**
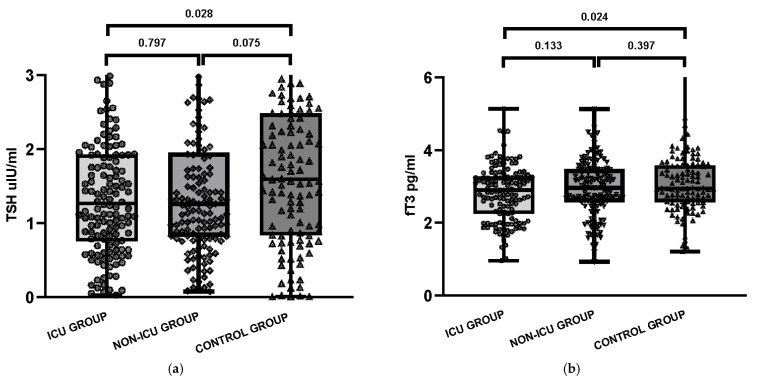

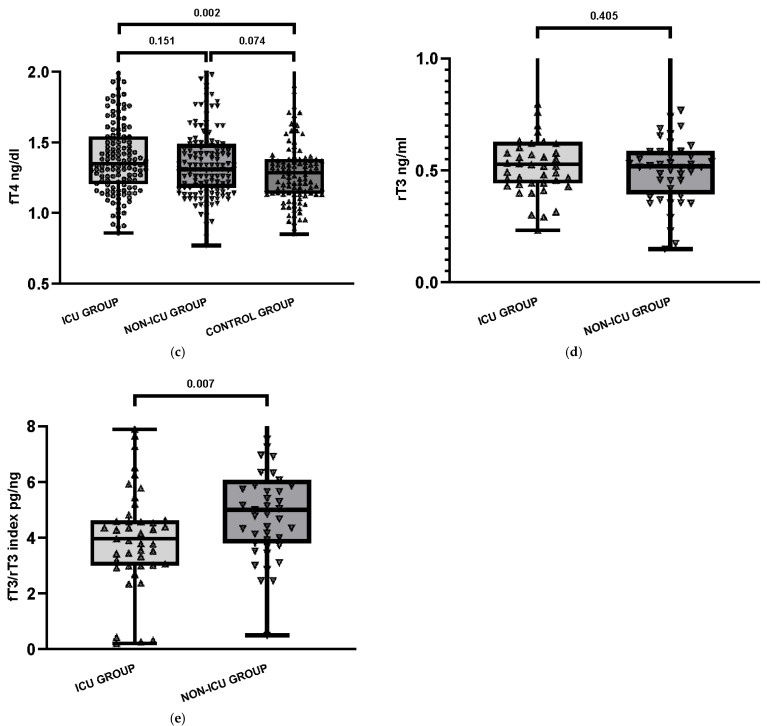
Serum concentrations of thyroid-related parameters in three study groups: COVID-19 patients admitted to the intensive care unit (ICU), COVID-19 patients hospitalized in non-ICU units, and the control group: (**a**) Thyroid-stimulating hormone (TSH); (**b**) Free triiodothyronine (fT3); (**c**) Free thyroxine (fT4); (**d**) Reverse triiodothyronine (rT3); (**e**) fT3/rT3 ratio.

Among ICU patients, 7.6% (*n* = 10) had low TSH levels (< 0.27 μIU/mL), while 6.5% (*n* = 9) of non-ICU patients showed similar findings. TSH serum levels were high (>4.20 μIU/mL) both in the COVID ICU group (2.9%, *n* = 4) and in COVID non-ICU group (3.6%, *n* = 5). No significant differences were observed between these two groups. Statistical analysis using the Kruskal–Wallis test showed no significant differences between the three groups for TSH (*p* = 0.151) and fT3 (*p* = 0.133), while a significant difference was observed for fT4 (*p* = 0.008). Baseline characteristics of the study groups are presented in Table 1 and illustrated in Figure 1.

The reverse T3 concentration was measured in the COVID-19 patient groups. The median rT3 level was 0.53 ng/mL in the COVID ICU group and 0.52 ng/mL in the COVID non-ICU group, both within the reference range (0.31–0.95 ng/mL). Reverse T3 was not assessed in the control group because the test was not ordered during routine clinical evaluation. Additionally, the calculated fT3/rT3 index was significantly lower in ICU patients (median 3.96 pg/ng) compared to non-ICU patients (median 5.00 pg/ng; *p* = 0.007), suggesting more pronounced impairment of peripheral thyroid hormone conversion, consistent with the biochemical profile of NTIS observed in critically ill individuals. Among the ICU group, 18 patients (13%) had low fT3 levels (<2.0 pg/mL), while 13 patients (9.4%) in the non-ICU group had similarly reduced fT3 values. These patients were further analyzed for the presence of thyroid autoantibodies. In the ICU subgroup with low fT3, six patients (33.3%) tested positive for at least one thyroid antibody, suggesting possible underlying autoimmune predisposition.

Compared with non-ICU patients, those admitted to the ICU more frequently had hypertension, diabetes mellitus, and coronary artery disease. A trend was also observed for chronic kidney disease. In contrast, obesity and heart failure did not significantly differ between ICU and non-ICU groups. In terms of treatment modalities (Table 1), ICU patients were more frequently treated with steroids (42.8% vs. 17.4%, *p* < 0.001), intubated (30.4% vs. 1.4%, *p* < 0.001), and received heparin (10.9% vs. 3.6%, *p* = 0.037). By contrast, there were no significant differences in non-invasive ventilation (CPAP/BiPAP; 18.1% vs. 16.7%, *p* = 0.874) or conventional oxygen therapy (nasal cannula or mask; 10.9% vs. 11.6%, *p* = 1.000).

**Figure 2:**
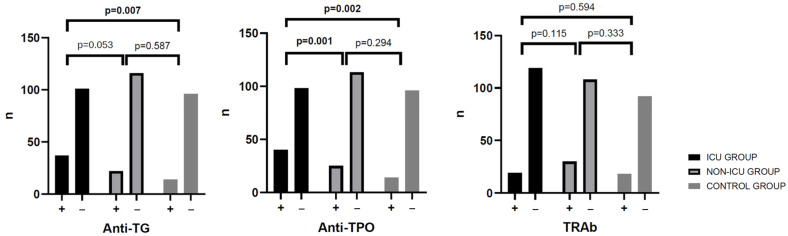
Presence of thyroid autoantibodies in ICU, non-ICU, and control groups.

In the non-ICU group, four of the 13 patients with low fT3 had detectable TRAb, and additional individuals had positive anti-TPO or anti-Tg antibodies. These findings emphasize that low fT3 was the most frequent abnormality among thyroid function tests, especially in the ICU group. Thyroid autoantibodies were detected in 39.1% of ICU patients (54/138) and 44.9% of non-ICU patients (62/138). Anti-TPO antibodies were present in 28.9% of COVID ICU patients, 25.0% of non-ICU patients, and 12.7% of controls. Isolated anti-TPO positivity (without co-occurrence of other antibodies) was observed in 7.3% and 1.4% of patients, respectively. Anti-Tg alone was positive in 4 (2.9%) and 8 (5.8%) participants in the COVID ICU and COVID non-ICU groups, respectively. Anti-TPO and anti-Tg were detected together in 15.2% of patients (*n* = 21) in the COVID ICU group and 17.4% of patients (*n* = 24) in the COVID non-ICU group. All variants of positive thyroid autoantibodies detected in participant groups are presented in Table 2 and Figure 2.

Significant differences in the distribution of thyroid autoantibody combinations were observed among the groups. The triple-positive variant (anti-Tg + anti-TPO + TRAb) was more common in COVID-19 patients, particularly in the non-ICU group (*p* = 0.009). Dual anti-Tg + anti-TPO positivity (*p* = 0.031) and anti-TPO only (*p* = 0.018) were also more frequent in COVID groups than in controls.

For the purpose of these analyses, 138 and 138 study participants were divided into eight subgroups based on their thyroid function: euthyroidism, clinical hyperthyroidism, subclinical hyperthyroidism, clinical hypothyroidism, subclinical hypothyroidism, low fT3 level, isolated elevated fT4 and isolated low fT4. Forty-three patients from COVID ICU group and thirty-nine from COVID non-ICU group had abnormal thyroid function test in acute COVID-19 (Table 3).

Five patients (2 from COVID ICU and 3 from COVID non-ICU group) had abnormal TFTs suggestive of subclinical thyrotoxicosis (low TSH with normal fT4 and fT3) during acute COVID-19. None of the five patients were positive for anti-TPO or anti-Tg antibodies. One patient had a mildly elevated TRAb level (2.33 IU/L). Among the 18 patients with low fT3 from COVID ICU group 6 patients had positive thyroid autoantibodies (3 patients aTg +, aTPO +, TRAb +, 2 patients aTg +, 1 patient aTg/aTPO +) when suffering from acute COVID-19, suggesting possible pre-existing thyroid autoimmunity. In the COVID non-ICU group 13 patients had low fT3 (four of them had positive TRAb, 2 patients aTg +, 1 patient aTg/aTPO + and 1 aTPO +). Two patients from COVID non-ICU group and one patient from COVID ICU group had subclinical hypothyroidism. All two of COVID non-ICU group had positive anti-TPO and anti-Tg status, consistent with a possible pre-existing Hashimoto’s thyroiditis.

Thyroid function parameters were compared across the COVID ICU, COVID non-ICU, and Control groups using Student’s *t*-test or Mann–Whitney U test for continuous variables, and the Chi-squared test or Fisher’s exact test for categorical variables. No significant differences were observed between the groups in terms of age and sex. Serum TSH (*p* = 0.028) levels were significantly lower in the COVID ICU group than in the Control group (non-COVID, non-ICU). Serum fT4 (*p* = 0.002) level was significantly higher in the COVID ICU group than in the Control group. The Kruskal–Wallis test showed that there was a statistically significant difference in anti-TPO among the groups (*p* = 0.001), but there was no significant difference in the median concentration of anti-Tg and TRAb between the COVID ICU and the COVID non-ICU groups. The median concentrations of anti-TPO, anti-Tg and TRAb in the participants was significantly higher than that of the participants in the Control group without inflammation and without COVID-19. In an exploratory analysis including age (per 10 years), sex, ICU status, and the fT3/rT3 index, none of these variables were significant predictors of thyroid autoantibody positivity (Table 4). When comorbidities were grouped into a binary variable (presence of ≥1 vs. none), no significant association was observed (OR 0.58, 95% CI 0.26–1.29, *p* = 0.19).

The association of NTIS and thyroid autoantibody positivity with in-hospital mortality was evaluated. In ICU patients, NTIS was not significantly related to mortality, while antibody positivity showed a borderline association. In the non-ICU group, NTIS showed no association, whereas antibody positivity was significantly associated with mortality (Table 5).

## 4. Discussion

This study demonstrates that thyroid function abnormalities and the presence of thyroid autoantibodies are common in patients hospitalized with COVID-19, particularly among those admitted to the ICU. We observed that 31.2% of ICU patients and 28.3% of non-ICU patients had thyroid function disturbances, with low fT3 being the most frequent finding. These results are consistent with the non-thyroidal illness syndrome (NTIS) pattern, which has been frequently reported in critically ill patients and during systematic inflammatory states such as sepsis or trauma [18].

Our findings confirm earlier reports that low serum fT3 levels, even in the presence of normal TSH, are common in severe COVID-19. Fliers et al. [4] and Sciacchitano et al. [18] previously described NTIS as a biochemical adaptation on critical illness but also noted its association with poor prognosis. In our cohort, ICU patients had significantly lower fT3 and higher fT4 levels compared to controls, which aligns with the pattern described in other studies, such as the THYRCOV study by Lania et al. [8], which reported NTIS in up to 70% of ICU patients with COVID-19. Prospective Chinese data showed that simultaneous low TSH and low fT3 independently predicted ICU mortality [7,19]. Similar prognostic value was observed in an Israeli ICU cohort [20] and in a Brazilian study where low fT3 at admission increased mortality risk seven-fold [21].

Interestingly, our ICU patients also had significantly higher fT4 levels than controls, which may reflect altered peripheral conversion of thyroid hormones or the effects of medication such as glucocorticoids or heparin, known to interfere with thyroid hormone transport or binding [22]. A similar pattern was described by Baldelli et al. [22], who observed elevated fT4 in patients receiving intensive treatment, although not all studies confirmed this trend. Similar findings were reported in a Turkish ICU cohort, where 42% of critically ill patients exhibited NTIS with suppressed TSH [23].

In contrast to some studies that reported low or suppressed TSH levels in ICU patients [24], we did not observe a statistically significant difference in TSH levels between the groups, although median TSH was lower in ICU patients. This discrepancy may be due to different time points of sample collection, disease severity, or medication use (e.g., corticosteroids, dopamine). Our finding more closely resembles those of Scappaticcio et al. [25], who reported similar TSH values across groups.

Our findings confirm previous large cohort studies reporting that hypertension, diabetes mellitus, and cardiovascular disease are the most common comorbidities associated with severe COVID-19 [26,27]. In contrast to some reports highlighting obesity as a major risk factor for severe COVID-19 [28], in our cohort obesity did not significantly differ between ICU and non-ICU patients. This discrepancy may reflect population-specific differences in obesity prevalence or the stronger impact of other comorbidities such as hypertension and coronary artery disease in our group.

In addition, the clinical characteristics of our study confirmed that ICU patients were characterized by more severe illness, as reflected by the higher frequency of invasive ventilation, steroid treatment, and heparin use. While non-invasive ventilation and conventional oxygen therapy were similarly used in both groups, this overlap reflects their broad application outside the ICU during the pandemic. Intensive Care Unit admission was mainly driven by the need for invasive ventilation, advanced monitoring, and the management of multi-organ failure risk. This finding aligns with the expectation that thyroid abnormalities observed in our study coexist with indicators of systemic disease severity.

Importantly, thyroid autoantibodies were significantly more frequent in COVID-19 patients than in the control group, with anti-TPO antibodies present in 28.9% of ICU patients and 25.0% of non-ICU patients, compared to 12.7% in controls. This pattern supports the hypothesis of either pre-existing thyroid autoimmunity or COVID-19-induced immune dysregulation. Persistent auto-antibody production has also been demonstrated up to 12 months post-infection [29]. Previous reports by Ruggeri et al. [6] and Rotondi et al. [30] documented the occurrence of subacute thyroiditis and antibody-positive thyroiditis in SARS-CoV-2 patients, suggesting that the virus can trigger autoimmune phenomena.

In our study, we observed statistically significant differences in the distribution of thyroid autoantibody patterns across the three groups. The triple-positive combination (anti-Tg + anti-TPO + TRAb) was more frequent among COVID-19 patients, particularly in the non-ICU group (*p* = 0.009). Similarly, dual anti-Tg + anti-TPO positivity (*p* = 0.031) and isolated anti-TPO positivity (*p* = 0.018) were significantly more common in the COVID-19 groups compared to controls. These findings support the notion of enhanced thyroid-specific autoimmune reactivity in SARS-CoV-2 infection, even in the absence of overt thyroid dysfunction. Our findings are consistent with the report by Rossini et al. [31], who found anti-TPO and anti-Tg positivity in 15.7% and in 6.7% of COVID-19 patients, compared with 7.7% and 8.0% in controls, respectively. In our study, anti-TPO and anti-Tg positivity was higher (anti-TPO: 28.9% in ICU and 25.0% in non-ICU; 12.7% in controls; anti-Tg: 24.6% in ICU and 27.1% in non-ICU; 3.6% in controls), possibly reflecting differences in patient populations and the higher proportion of critically ill individuals.

Such associations between thyroid autoantibodies and thyroid dysfunction have been reported previously [32], and our findings further support the link between autoimmune activity and altered thyroid function in the context of COVID-19.

The high rate of combined anti-TPO and anti-Tg positivity in our study further supports a potential link to Hashimoto-s thyroiditis. However, the frequency of overt hypothyroidism or hyperthyroidism remained low. This suggests that most observed thyroid dysfunctions were functional rather than destructive or autoimmune in origin. Our findings are in agreement with previous reviews [33], which reported increased prevalence of thyroid antibodies in COVID-19 without corresponding rates of clinical thyroid disease.

Our analysis did not identify significant predictors of thyroid autoantibody positivity among COVID-19 patients. Neither demographic factors, ICU status, thyroid hormone indices, nor comorbidity burden (≥1 vs. none) showed significant associations. This suggests that antibody presence may be driven more by individual predisposition or post-infectious immune activation rather than baseline clinical characteristics.

Another key observation was the significant decrease in the fT3/rT3 index in ICU patients, a sensitive marker of NTIS. This supports the concept that impaired peripheral deiodination, possibly due to cytokine-induced inhibition of type 1 deiodinase, plays a central role in thyroid hormone imbalance in COVID-19 [13,18].

Although reverse T3 levels in our cohort remained within the reference range, the significantly lower fT3/rT3 index in the ICU patients likely reflects impaired peripheral deiodination rather than absolute rT3 elevation. This observation aligns with the known inhibitory effect of proinflammatory cytokines on type 1 deiodinase, which converts T4 to active T3. Previous studies have shown that the fT3/rT3 ratio is a more sensitive indicator of altered thyroid hormone metabolism in critical illness than fT3 or rT3 alone, and may better reflect the severity of systematic illness and prognosis [7,18]. The presence of low fT3 in 13% of ICU patients and 9.4% of non-ICU patients further supports the clinical relevance of NTIS in COVID-19, in line with prior studies highlighting its prevalence and prognostic value [8,19,21]. The presence of low fT3 without overt hypothyroidism suggests a functional and potentially reversible adaptation to critical illness, rather than structural thyroid gland damage.

Interestingly, several patients with positive autoantibodies had isolated low fT3, but did not meet criteria for hypothyroidism. Whether this reflects a predisposition to more severe NTIS in patients with pre-existing thyroid autoimmunity remains unclear. Our findings partially support the hypothesis by Muller et al. [34], who proposed that autoimmune thyroid disease may influence COVID-19 outcomes through immune dysregulation [34].

Encouragingly, most thyroid function abnormalities resolved within 12 weeks of recovery, as demonstrated in a longitudinal UK cohort study [35]. Furthermore, a meta-analysis of 30 studies confirmed that patients with severe COVID-19 have an approximately fourfold increased risk of abnormal thyroid function tests [36]. A recent narrative review has comprehensively summarized the evolving relationship between COVID-19 and thyroid dysfunction, highlighting the complexity and multifactorial nature of this association [37].

Our work adds to the literature by a) directly comparing ICU and non-ICU COVID-19 groups with a non-COVID control group, which has rarely been addressed in previous studies, b) examining combined thyroid autoantibody profiles (showing that the triple-positive pattern remains significant even after Bonferroni adjustment), and c) evaluating the fT3/rT3 ratio as a severity-linked marker of NTIS. Together, these findings refine the understanding of how thyroid status and autoimmunity relate to COVID-19 severity.

In our study, NTIS did not consistently predict mortality, supporting its role as a marker of acute disease severity rather than an independent prognostic factor. In contrast, thyroid autoantibody positivity was associated with increased mortality outside the ICU, suggesting that underlying autoimmunity may contribute to poorer outcomes in non-ICU patients.

These findings underscore the importance of ongoing monitoring of thyroid function in patients recovering from COVID-19, particularly those with severe illness or abnormal test results during the acute phase.

The limitations should be acknowledged in our investigation. Most importantly, the thyroid status of patients prior to SARS-CoV-2 infection was not definitively known, which limits the ability to fully determine whether the observed abnormalities were newly developed during acute illness or reflected pre-existing dysfunction. Nonetheless, to minimize this uncertainty, medical documentation was thoroughly reviewed, and only patients with no recorded thyroid diseases in their medical history were included in the analysis.

Another limitation of our study is that thyroid function tests were not performed routinely in all hospitalized patients but only when clinically indicated, which may introduce a selection bias. To reduce this risk, we included only patients with a complete panel of thyroid hormones and autoantibodies. Moreover, the control group was carefully selected to exclude infectious etiologies, as all individuals had negative SARS-CoV-2 PCR results, low procalcitonin levels (<0.5 ng/mL), and no clinical signs of infection such as fever or cough. This design allowed us to compare COVID-19 with non-infectious conditions. However, while our findings indicate that thyroid alterations are associated with COVID-19, we cannot exclude the possibility that similar changes may also occur in the context of other acute infections. Lack of assessment of variant-specific effects is also a limitation, since routine genomic sequencing was not performed in clinical practice at that time. Furthermore, a considerable proportion of ICU patients died during hospitalization, often shortly after admission, which restricted the possibility of evaluating variant-related outcomes. Finally, no adjustment for multiple comparisons was applied; therefore, results should be interpreted with caution. Notably, the association with triple-positive antibody status remained statistically significant even after Bonferroni correction (adjusted threshold *p* < 0.01).

## 5. Conclusions

In conclusion, our results confirm that thyroid dysfunction is common in COVID-19, especially in ICU patients. The NTIS pattern, elevated prevalence of thyroid autoantibodies, and altered fT3/rT3 ratio all indicate that thyroid status mirrors both disease severity and immune activation. These findings support the role of thyroid assessment in clinical risk stratification, while further longitudinal studies are needed to determine the long-term impact of COVID-19 on thyroid function and autoimmunity.

## Figures and Tables

**Table 1:** Baseline characteristics of the COVID ICU patients, the COVID non-ICU patients and Control group.

Variable	COVID ICU *n* = 138	COVID Non-ICU *n* = 138	Control Group *n* = 110	*p*-Value (ICU vs. Non-ICU)	*p*-Value (ICU vs. Control)	*p*-Value (Non-ICU vs. Control)
Age (years), median [Q1–Q3]	70 [58–80]	67 [53–78]	50 [36–69]	0.197	<0.001	<0.001
Male sex, *n* (%)	56 (40.6%)	54 (39.1%)	30 (27.3%)	0.902	0.032	0.059
Female sex, *n* (%)	82 (59.4%)	84 (60.9%)	80 (72.7%)			
TSH, median [Q1–Q3]	1.27 [0.77–1.93]	1.26 [0.82–1.94]	1.59 [0.84–2.46]	0.797	0.028	0.075
fT4, median [Q1–Q3]	1.35 [1.21–1.54]	1.31 [1.18–1.49]	1.29 [1.14–1.38]	0.151	0.002	0.074
fT3, median [Q1–Q3]	2.90 [2.26–3.26]	2.96 [2.57–3.47]	2.95 [2.58–3.57]	0.133	0.024	0.397
rT3, median [Q1-Q3]	0.53 [0.44–0.62]	0.52 [0.40–0.58]	-	0.405	-	-
fT3/rT3 index (pg/ng)	3.96	5.00	-	0.007	-	-
% anti-TPO (+)	28.9%	25.0%	12.7%	0.001	0.002	0.294
% anti-Tg (+)	24.6%	27.1%	3.6%	0.053	0.007	0.587
% TRAb (+)	17.4%	24.3%	16.4%	0.115	0.594	0.333
Comorbidities						
Hypertension, *n* (%)	32 (23.2%)	13 (9.4%)	-	0.003	-	-
Diabetes mellitus, *n* (%)	19 (13.8%)	8 (5.8%)	-	0.041	-	-
Obesity, *n* (%)	24 (17.4%)	17 (12.3%)	-	0.310	-	-
Heart failure, *n* (%)	7 (5.1%)	3 (2.2%)	-	0.335	-	-
Coronary artery disease, *n* (%)	24 (17.4%)	10 (7.2%)	-	0.016	-	-
Chronic kidney disease, *n* (%)	9 (6.5%)	2 (1.4%)	-	0.060	-	-
Clinical condition						
Oxygen therapy (nasal cannula/mask), *n* (%)	15 (10.9%)	16 (11.6%)	-	1.000	-	-
Non-invasive ventilation (CPAP/BiPAP), *n* (%)	25 (18.1%)	23 (16.7%)	-	0.874	-	-
Intubation, *n* (%)	42 (30.4%)	2 (1.4%)	-	<0.001	-	-
Steroid treatment, *n* (%)	59 (42.8%)	24 (17.4%)	-	<0.001	-	-
Heparin treatment, *n* (%)	15 (10.9%)	5 (3.6%)	-	0.037	-	-

Reference range: TSH μIU/mL (0.270–4.200), fT4 ng/dL (0.92–1.68), fT3 pg/mL (2.0–4.4), rT3 ng/mL (0.31–0.95). Abbreviations: CPAP—continuous positive airway pressure; BiPAP—bilevel positive airway pressure.

**Table 2:** Frequency of thyroid autoantibody combinations in the study groups. Statistically significant differences between groups were identified using the chi-square test (*p*-values shown).

Thyroid Autoantibodies	COVID ICU Group *n* = 138	COVID Non-ICU Group *n* = 138	Control Group *n* = 110	*p* (χ^2^ Test )
Anti-Tg +, anti-TPO +, TRAb +	13 (9.4%)	24 (17.4%)	6 (5.5%)	0.009
Anti-Tg +, anti-TPO +	21 (15.2%)	24 (17.4%)	7 (6.4%)	0.031
Anti-Tg +	4 (2.9%)	8 (5.8%)	1 (0.9%)	0.098
Anti-TPO +	10 (7.2%)	2 (1.4%)	2 (1.8%)	0.018
TRAb +	6 (4.3%)	4 (2.9%)	2 (1.8%)	0.514

**Table 3:** Thyroid function among COVID ICU and COVID non-ICU patients.

	COVID ICU Group (*n* = 138)	COVID Non ICU Group (*n* = 138)	*p* (χ^2^ Test)
Normal thyroid function test	95 (68.8%)	99 (71.7%)	0.423
Low fT3	18 (13.0%)	13 (9.4%)	0.343
Isolated elevated fT4	12 (8.7%)	7 (5.1%)	0.241
Subclinical thyrotoxicosis	2 (1.4%)	3 (2.2%)	0.652
Overt thyrotoxicosis	6 (4.3%)	9 (6.5%)	0.430
Subclinical hypothyroidism	1 (0.7%)	2 (1.4%)	0.561
Overt hypothyroidism	3 (2.2%)	4 (2.9%)	0.704
Isolated low fT4	1 (0.7%)	1 (0.7%)	1.000

**Table 4:** Logistic regression and univariable analysis of predictors for thyroid autoantibody positivity (≥1 of anti-TPO, anti-Tg, TRAb) among COVID-19 patients.

Predictor	OR	95% CI	*p*-Value
Age (per 10 years)	1.12	0.85–1.49	0.415
Male sex (vs. female)	0.94	0.38–2.31	0.889
ICU (vs. non-ICU)	0.83	0.33–2.07	0.689
fT3/rT3 index (per 1)	1.02	0.86–1.21	0.823
≥1 comorbidity (vs. none) *	0.58	0.26–1.29	0.190

* Univariable Fisher’s exact test.

**Table 5:** Association of NTIS and thyroid autoantibody positivity with in-hospital mortality.

Group	Predictor	Deaths with Predictor *n* (%)	Survivors with Predictor *n* (%)	Deaths Without Predictor *n* (%)	Survivors Without Predictor *n* (%)	OR	95% CI
ICU	NTIS	11 (36.7)	19 (63.3)	29 (26.9)	79 (73.1)	1.58	0.67–3.71
	Any Ab +	21 (38.2)	34 (61.8)	19 (22.9)	64 (77.1)	2.08	0.99–4.39
non-ICU	NTIS	2 (10.0)	18 (90.0)	13 (11.1)	104 (88.9)	0.89	0.19–4.28
	Any Ab +	10 (21.7)	36 (78.3)	5 (5.5)	86 (94.5)	4.78	1.53–14.97

OR—odds ratio; CI—confidence interval; NTIS defined as fT3 < 3.1 pg/mL. Any Ab + = positivity for ≥1 of anti-TPO, anti-Tg, or TRAb. Fisher’s exact test.

## Data Availability

The data presented in this study are available on request from the corresponding author.

## Abbreviations

The following abbreviations are used in this manuscript:

Anti-Tg	Anti-thyroglobulin antibody
Anti-TPO	Anti-thyroid peroxidase antibody
BiPAP	Bilevel positive airway pressure
COVID-19	Coronavirus Disease 2019
CPAP	Continuous positive airway pressure
fT3	Free triiodothyronine
fT4	Free thyroxine
HPT axis	Hypothalamic–pituitary–thyroid axis
ICU	Intensive Care Unit
IQR	Interquartile range
NTIS	Non-thyroidal Illness Syndrome
rT3	Reverse triiodothyronine
TFTs	Thyroid function tests
TRAb	Thyrotropin receptor antibody
TSH	Thyroid-stimulating hormone
